# Advancing cell therapies with artificial intelligence and synthetic biology

**DOI:** 10.1016/j.cobme.2025.100580

**Published:** 2025-02-03

**Authors:** Mahima Choudhury, Annika J. Deans, Daniel R. Candland, Tara L. Deans

**Affiliations:** a Department of Biomedical Engineering, University of Utah, Salt Lake City, UT, USA; b Department of Biomedical Engineering, University of Rochester, Rochester, NY, USA; c Wallace H. Coulter Department of Biomedical Engineering, Georgia Institute of Technology and Emory University, Atlanta, GA, 30332, USA

**Keywords:** Artificial intelligence, Machine learning, Cell therapy, Synthetic biology

## Abstract

Artificial intelligence provides an exciting avenue to improve approaches in cell therapies by learning and predicting dynamic gene expression patterns from large datasets of stem cell differentiation. The integration of synthetic biology provides genetic tools that mimic the spatial and temporal expression patterns during differentiation, enhancing the potential to significantly improve differentiation outcomes and further our understanding of the mechanisms involved during cell fate decisions.

## Introduction

Regenerative medicine is a cell therapy that focuses on harnessing the body’s own capabilities to repair, replace, or regenerate damaged tissues and organs [1]. However, some cells have limited or no capacity to regenerate making it difficult for the body to naturally repair damaged tissue. Stem cells have the potential to play an important role in augmenting the healing process, however directing their cell fate has proven to be difficult in standard cultures [2,3]. Strategies to direct stem cell differentiation into desired lineages traditionally include adding growth factors to the culture medium in a stepwise fashion. This method often does not consider that the development of organs and tissues naturally involves distinct gene expression patterns which are controlled spatially and temporally by the organism, resulting in low yields of desired cell populations and heterogeneity among cells in cultures [4,5]. Furthermore, scaling up stem cell differentiation for therapeutic applications is difficult using growth factors due to substantial lot-to-lot variability which limits reproducibility, in addition to their considerable cost. Finding new approaches to better understand cell fate decisions and to control stem cell differentiation to produce high yields of the desired cell population will be crucial to improve clinical outcomes.

Gene regulatory networks (GRNs) are collections of genes and regulatory proteins that interact with each other within cells to produce specific functions and are essential in controlling cell fate decisions that include self-renewal, differentiation, and apoptosis [6]. Within these GRNs are cis-regulatory proteins that control the expression of gene encoding transcription factors in a spatial and temporal manner. The expression of these transcription factors is essential for cell fate decisions, however decoding how cells use this information to orchestrate gene expression patterns to generate complex tissues has been challenging [7].

**Figure 1:**
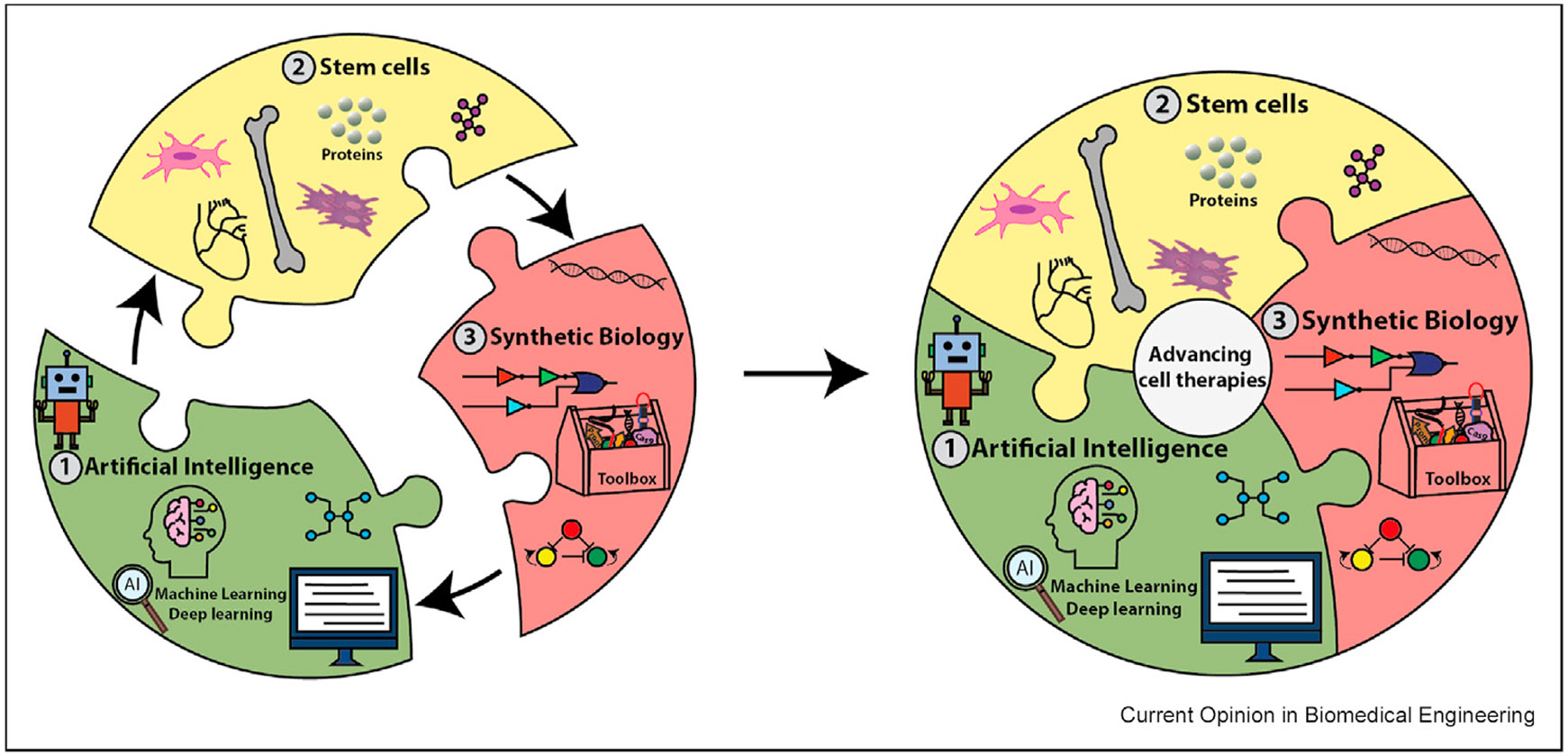
Advancing cell therapies with AI and synthetic biology. AI can be used to better understand stem cell fate decisions to produce a roadmap for building new synthetic gene circuits to direct stem cell differentiation for a variety of cell therapy applications.

To improve cell therapy approaches, we believe that computational models can be utilized to identify relationships between gene expression patterns at any given time during the cell fate process. This will streamline the construction of sophisticated genetic tools which can be used to direct stem cell differentiation into desired lineages either *in vitro* or *in vivo*. In this review, we discuss the need for understanding the mechanisms and dynamics of gene expression that take place during stem cell differentiation to improve clinical outcomes in cell therapies. We highlight stem cell differentiation studies that show how changing the expression of transcription factors can drive profound changes in cell fate, along with recent studies that use AI to predict stem cell identity and behavior. Finally, we discuss how AI can accelerate the design of synthetic gene circuits to tightly regulate the expression of transcription factors for enhancing stem cell fate decisions to improve clinical outcomes (Fig. 1). Interfacing these three fields will lead to a better understanding of the mechanisms of stem cell differentiation, tissue formation, and enabling the programming of cells to enhance robust differentiation into desired cell lineages.

### Stem cells

**Figure 2:**
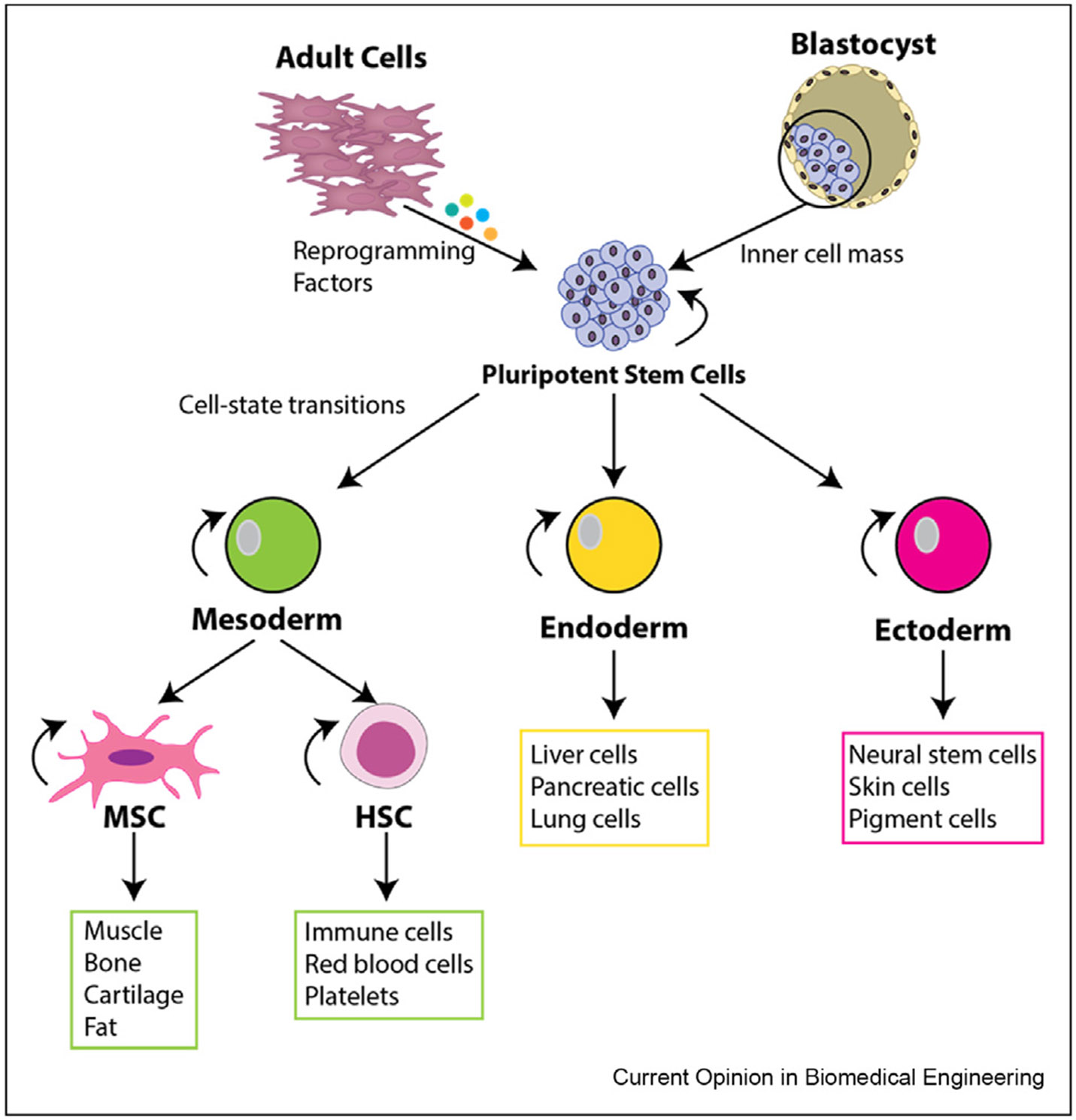
Stem cell differentiation. Pluripotent stem cells can be derived from adult cells by using reprogramming factors or from harvesting the inner cell mass from a blastocyst. Pluripotent stem cells (purple) have the potential to be any cell in the body. These stem cells can form all three germ layers: mesoderm (green), endoderm (yellow), and ectoderm (pink). Stem cells in each germ layer can continue differentiating to give rise to different organs and tissues in the body. Cell-state transitions occur as the cell moves from one defined cell to another. Stem cells self-renew (arrow going up) to maintain populations for replenishing and repairing adult cells. Mesenchymal stem cell (MSC), hematopoietic stem cell (HSC).

Stem cells are unspecialized cells that have the capacity to self-renew as well as differentiate into mature cell types that serve as a repair system for the body. Pluripotent stem cells have the capacity to differentiate into all somatic cells in the body, making them an ideal cell source for many cell therapy applications [8]. During development, one of the first cell fate decisions to be made by pluripotent stem cells is the specialization into one of the three embryonic germ layers: ectoderm, mesoderm, and endoderm, which give rise to multipotent stem cells [9-11]. From there, cells activate and repress various transcription factors to further their cell commitment down various lineages (Fig. 2).

Transcription factors control the expression of genes by binding to specific DNA sequences throughout the genome and can dynamically regulate pathways to induce cellular states. For example, the overexpression of Oct4, Sox2, Klf4, and c-myc in adult cells can override previously made cell fate decisions to reprogram them into a pluripotent state [12]. These induced pluripotent stem cells (iPSCs) have the potential to become any somatic cell type in the body, making it possible to treat diseased and damaged tissues by replacing lost or damaged cells with patient-specific stem cells. Alternatively, as cells receive the signals to differentiate, the expression of these pluripotent transcription factors turn off, and other transcription factors turn on, initiating the cells to differentiate to become more specialized.

Tremendous progress has been made in driving stem cell fate by the ectopic overexpression of transcription factors with the rationale that this overexpression triggers cell transitions to drive stem cell differentiation. Studies have confirmed that the overexpression of transcription factors in pluripotent stem cells can direct their differentiation toward many different cell types [13,14] including microglia [15,16], hematopoietic stem cells [17], hepatocytes [18], and megakaryocytes [19]. However, many of these methods have shown that heterogeneous cell populations including functionally immature cells are not sufficient for clinical use [20].

Improved approaches to better understand the role of transcription factors on GRNs and how their expression is used to control cellular programs to direct stem cell differentiation has been the focus of many studies. Recently, a barcoded library of all annotated human transcription factor splice isoforms was created and used in pluripotent stem cells to build a library and Atlas for studying the effects of transcription factor overexpression [21]. This work identified bottlenecks in cell fate specifications and suggests which transcription factors may drive pluripotent stem cells toward specific cell fates, providing exciting opportunities for cell engineering approaches.

### Computational approaches and artificial intelligence

Computational approaches have proven to be powerful tools for analyzing large data sets to accurately characterize biological processes and predict behavior [22-25]. Artificial Intelligence (AI) was initially developed to improve the speed, accuracy, and precision of performing complex tasks that was incorporated in technologies such as speech and facial recognition [26,27]. More recently, machine learning (ML) and deep learning (DL) have advanced AI in the scientific and medical communities which learn the relationship between input and output data and can be used to predict cell classifications [28], identify transcription factors in biological processes [20], better detect infectious diseases and drug discoveries [29], enzyme engineering [30], and much more. ML is an area of AI that focuses on statistical models that have accelerated scientific discovery and enabled a deeper understanding of complex biological phenomena by analyzing large publicly available datasets to extract insights and make informed decisions based on the given dataset. Many reviews extensively discuss the different types of ML and DL models, their inputs-outputs, and how each model processes data [31-33]. Here we discuss how AI is being used to improve cell manufacturing and stem cell classification, two important areas for improving cell therapies.

### Cell manufacturing

Scalable manufacturing strategies are required to translate stem cells and engineered cells to the clinic for treating injured and diseased tissues. Since iPSCs have the potential to differentiate into any cell type in the body, they are the ideal cell type to manufacture for supporting the most clinical opportunities for regenerative medicine and cell therapies. Traditionally, when iPSCs are grown *in vitro*, they form colonies and adhere to the tissue culture dish, a method that is not conducive to large-scale manufacturing. To manufacture large numbers of cells, stirred suspension cultures are the preferred method, however iPSCs have the tendency to cluster, which ultimately impacts the distribution of nutrients to cells. This uneven distribution of nutrients to cells can result in heterogeneous cell populations and cell death [34-36]. Recently a new AI model, biological systems-of-system (Bio-SoS), was introduced to better understand and predict the most successful cell manufacturing methods for iPSC growth and expansion [37]. The construction of this model included training data from a collection of monolayer cells and cell aggregates. This model accurately predicted the metabolic dynamics and heterogeneity in various cell aggregates that was validated with experiments. More broadly, using AI to analyze data from cell cultures in real-time from bioreactors and large-scale cell cultures to monitor nutrient levels, temperature, and gas levels can optimize growth conditions to increase cell yield for maximizing cell manufacturing.

### Stem cell classification

Computational tools have been used to help identify cell features to accurately classify subpopulations of stem cells that may be difficult to identify otherwise [38-43]. Hematopoietic stem cells (HSCs) reside in the bone marrow and give rise to all cells in the blood system through hematopoiesis, an extensive and complex process that involves cell fate branching, bypassing, and feedback loops to reach the final adult cell [44-46]. This complexity has made it difficult to identify and isolate transient progenitor stem cells that can offer insights to which cell populations should be harvested to improve cell transplantations, and which cells lead to the development of disease. Recently, DL was used to distinguish between HSCs and multipotent progenitors (MPPs) solely based on their morphology using light microscopy images [47]. This method was able to detect specific cell clusters and classify input images that identified different progenitor cell types.

Similar work has been done with mesenchymal stem cells [48]. Mesenchymal stem cells (MSCs) have the capacity to differentiate into various cells of the musculoskeletal system and have a high immunomodulatory influence that has led to their use in preclinical and clinical phases to treat various diseases [49]. In this study, the goal was to identify a level of quality control of MSCs to provide consistent, high-quality, large-scale biomanufacturing of MSCs for successful clinical translation. The DL framework introduced in this study enables a non-invasive and high throughput method for screening MSCs to improve biomanufacturing MSCs for clinical use.

While not all labs have the expertise to seamlessly implement computational approaches in their work, a recent platform, BioAutoMATED, was introduced to improve accessibility for analyzing large data sets and implementing AI to scientists who may not already have the expertise to build their own custom ML models [50]. BioAutoMATED takes basic biological inputs such as DNA, RNA, amino acid sequences, and glycans of any length, type, or function, and proceeds without any additional user intervention. This platform provides a promising avenue for scientists to integrate ML into their workflow and has been verified in gene regulation, antibody-drug binding, glycan immunogenicity and classification, and toehold switch design.

### Synthetic biology

Synthetic biologists design and build genetic tools called synthetic gene circuits that consist of an assembly of gene regulatory parts to be used for programming cells with new functions. These tools can also be used to repurpose cellular pathways for programming cells with customized input–output behavior. Advances in genome editing and synthetic gene synthesis have accelerated our ability to build synthetic gene circuits that tightly regulate gene expression in cells. This has transformed our ability to program cells to tackle complex disease conditions including infectious disease treatments, immunotherapy, metabolic disorders, and cancer [51-54]. Synthetic biology has produced several genetic tools to control gene expression with various dynamic levels of control that include feedback, switches, oscillators, pulse generators, and Boolean logic functions [55-64]. Synthetic gene circuits are poised to program stem cells to tightly control gene expression spatially and temporally for directing stem cell differentiation with a high degree of programmability.

### AI for accelerating synthetic gene circuit design

Since its inception two decades ago, synthetic biology has revolutionized how cells can be programmed with new functions by assembling gene regulatory parts [65]. Building synthetic gene circuits typically begins with a desired functional input–output behavior and genetic parts are assembled to follow the Design-Build-Test-Learn (DBTL) paradigm. The choice of promoter strength, binding strength of regulatory proteins, and the half-life of proteins used are all critical parameters to consider in the synthetic gene circuit design. Additionally, genetic parts can be built in different combinations to explore output behaviors within the cell. While the DBTL approach enables continuous quality improvements of circuit function, genetic circuits often need to be tuned to meet specifications for the desired application, and this cycle can take a few, and sometimes many iterations before the circuit is functioning in the desired way in cells. This process can take time, creating a bottleneck for programming cells and often delaying their widespread use in clinical and biotechnology settings. To address this challenge, efforts are underway to use data-driven computational approaches for designing synthetic gene circuits to program cells that can produce robust functional changes to perform in various microenvironments.

Engineering custom receptors for cells to sense extracellular molecules and produce a desired cellular response has become the focus of many synthetic biologists to engineer cells with a targeted therapeutic response at the site of disease [66-73]. The engineering of immune cells has been a primary focus of many studies, specifically T cells, with chimeric antigen receptors (CARs), to target and kill diseased cells [51,52,74]. While this approach has proven to be successful for sensing and responding to specific extracellular signals to eradicate diseases, designing gene circuits that encode for synthetic receptors can be time consuming, requiring dozens of engineered receptors to be built and tested in order to achieve tumor specificity and the appropriate cell activation to target and kill tumor cells. To address this bottleneck, a recent study demonstrated that machine learning can be used to predict gene circuit design and function, essentially rapidly iterating through the DBTL process and predicting which CAR designs would likely be the most effective [75]. Briefly, this study built a library of ~2400 engineered receptors containing multiple stimulatory domains that can be rearranged in different positions. These engineered receptors were transduced into T cells and tested for stemness and cytotoxicity, two important characteristics required for tumor clearance. The results from this study were used to train machine learning models to predict improved designs for engineered receptors [76].

Coupling AI and synthetic biology has the potential to transform our ability to build better synthetic gene circuits and to better understand cell fate mechanisms, providing a roadmap for programming stem cells to enable reproducible and robust differentiation outcomes for regenerative medicine applications.

### AI for road mapping synthetic gene circuit design for directing stem cell fate

Many cellular commitment pathways require complex signaling behaviors that include dynamic gene expression, and the temporal regulation of transcriptional activators and repressors [77,78]. Directing dynamic behaviors in a cell culture dish is difficult when adding growth factors to the culture medium. Tools in synthetic biology can tightly regulate transcription factor expression in many dynamic patterns which can provide a new approach to cell fate reprogramming. This would enable the expression of transcription factors at realistic biological levels in a spatial and temporal fashion instead of their continuous overexpression. The recent development of FateCompass estimates transcription factor dynamics during the differentiation from mouse pluripotent stem cells to pancreatic endocrine cells and offers exciting insights on how to build genetic tools to dynamically regulate cell fate [79]. Similarly, genetically encoded sensors were recently built to sense levels of distinct microRNAs in stem cell as they are differentiating (i.e. cell transition states) to regulate the expression of specific transcription factors to guide the stem cells to a desired lineage [80].

Our understanding of how gene regulation is coordinated within cells continues to improve with the evolution of computational approaches to analyze large datasets [81,82]. Specifically, understanding the dynamic gene expression patterns as stem cells move through differentiation, from one cell state to the next, and how these expression patterns ultimately drive cells to commit to a particular terminally differentiated adult cell will facilitate the building of new genetic tools to mimic these patterns to improve differentiation outcomes.

By analyzing differentiation data, AI has the potential to uncover gene expression patterns and key regulatory pathways not yet identified during the cell fate process, which can be used as a roadmap to build new synthetic gene circuits to control cell fate decisions. Taking hematopoiesis as an example, we imagine a workflow where differentiation data can be used to train an ML model and then identify distinct gene expression patterns for cells to either become B cells, macrophages, red blood cells, etc. Using this information, synthetic gene circuits can be designed and built with accelerated ML models to program progenitor cells to enhance their cell fate outcomes.

## Conclusion

The past two decades have seen tremendous discoveries in the stem cell field with the demonstration that adult stem cells can be reprogrammed to a pluripotent state, making it possible to use patient-specific cells for repairing damaged tissue to restore lost function. This review emphasizes the opportunities for AI to predict transcription factor expression patterns during differentiation that can provide a roadmap for synthetic biologists to build new genetic tools that direct stem cell differentiation into desired lineages. Exploring these opportunities will likely provide insights on how genes are expressed as cells transition from one stage to the next, and how changes to gene expression patterns can enhance desired differentiation outcomes.

## Data Availability

No data was used for the research described in the article.
